# Biosynthetic Pathway and Health Benefits of Fucoxanthin, an Algae-Specific Xanthophyll in Brown Seaweeds

**DOI:** 10.3390/ijms140713763

**Published:** 2013-07-02

**Authors:** Koji Mikami, Masashi Hosokawa

**Affiliations:** Faculty of Fisheries Sciences, Hokkaido University, 3-1-1 Minato-cho, Hakodate 041-8611, Japan; E-Mail: hoso@fish.hokudai.ac.jp

**Keywords:** biosynthetic pathway, brown seaweed, carotenoid, carotenogenic gene, *Ectocarpus siliculosus*, fucoxanthin, genome, health benefit

## Abstract

Fucoxanthin is the main carotenoid produced in brown algae as a component of the light-harvesting complex for photosynthesis and photoprotection. In contrast to the complete elucidation of the carotenoid biosynthetic pathways in red and green algae, the biosynthetic pathway of fucoxanthin in brown algae is not fully understood. Recently, two models for the fucoxanthin biosynthetic pathway have been proposed in unicellular diatoms; however, there is no such information for the pathway in brown seaweeds to date. Here, we propose a biosynthetic pathway for fucoxanthin in the brown seaweed, *Ectocarpus siliculosus*, derived from comparison of carotenogenic genes in its sequenced genome with those in the genomes of two diatoms, *Thalassiosira pseudonana* and *Phaeodactylum tricornutum*. Currently, fucoxanthin is receiving attention, due to its potential benefits for human health. Therefore, new knowledge regarding the medical and nutraceutical properties of fucoxanthin from brown seaweeds is also summarized here.

## 1. Introduction

Carotenoids are tetraterpenoids with a characteristic linear C_40_ molecular backbone containing up to 11 conjugated double bonds, which are produced in photosynthetic organisms, including seaweeds [1,2]. Most carotenoids are colorful pigments reflecting yellow, orange and red light, and their presence is responsible for color in flowers, fruits and vegetables. Animals cannot synthesize carotenoids; however, they absorb and accumulate carotenoids from their diets, which results in, for example, the pink and orange hues of lobster shells, salmon meat and flamingo feathers [1].

Carotenoids have a diverse range of functions in addition to coloration. In plants, they are essential components of the photosynthetic antenna and reaction center complex in thylakoid membranes of chloroplasts. Carotenoids are also involved in photosystem assembly and light harvesting for photosynthesis and also in the protection of the photosynthetic apparatus from photo-oxidative stress, due to high radical scavenging activity [3,4]. In addition, carotenoids are precursors of the plant hormone, abscisic acid [5,6], which is known to play roles in plant responses to abiotic stresses, such as desiccation and low temperature [7]. Moreover, strigolactone, a recently identified phytohormone involved in the inhibition of shoot branching, is also derived from carotenoids [8–10]. Thus, carotenoids are essential second metabolites in both photosynthetic and non-photosynthetic tissues in plants. Because of these important functions, the biosynthetic pathways of carotenoids have been extensively investigated in plants, fungi and phytoplankton through molecular cloning and expression analysis of carotenogenic genes [1,3,4].

**Figure 1:**
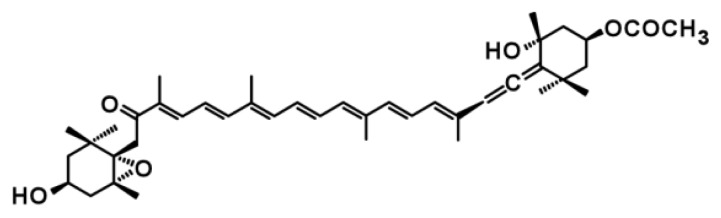
Molecular structure of fucoxanthin.

Seaweeds are marine photosynthetic organisms, whose carotenoid profiles are used as a basis for taxonomic classification into green, red and brown algae [2]. Seaweeds commonly contain β-carotene, a precursor of vitamin A that is absorbed from the diet and is required for normal growth and tissue repair in animals [11]. Recently, attention has been focused on fucoxanthin, because of its health benefits, such as antioxidant, anti-inflammation, anti-cancer and anti-obesity activities [12,13]. Fucoxanthin is found in brown seaweeds, diatoms and dinoflagellates and has a unique structure, including an allenic bond, an epoxide and a conjugated carbonyl group in the polyene chain of the molecule (Figure 1), which distinguishes its structure from that of plant carotenoids, such as β-carotene and lutein. However, in contrast to the complete elucidation of the carotenogenic pathway genes in green terrestrial plants [1,3,4], little is known regarding the biosynthetic pathway of fucoxanthin at either the biochemical or molecular biological levels, which hinders lower cost production of this carotenoid through biotechnological approaches. In addition, to our knowledge, the biosynthetic pathways of fucoxanthin in brown seaweeds have not been published, although hypothetical pathways have been proposed for diatoms [14–16]. Therefore, we summarize here the current status of the understanding of fucoxanthin biosynthesis in brown seaweeds and present the state of knowledge regarding the biological functions of fucoxanthin in human, animal and mammalian cell culture.

## 2. Presence of Fucoxanthin in Brown Seaweed

**Figure 2:**
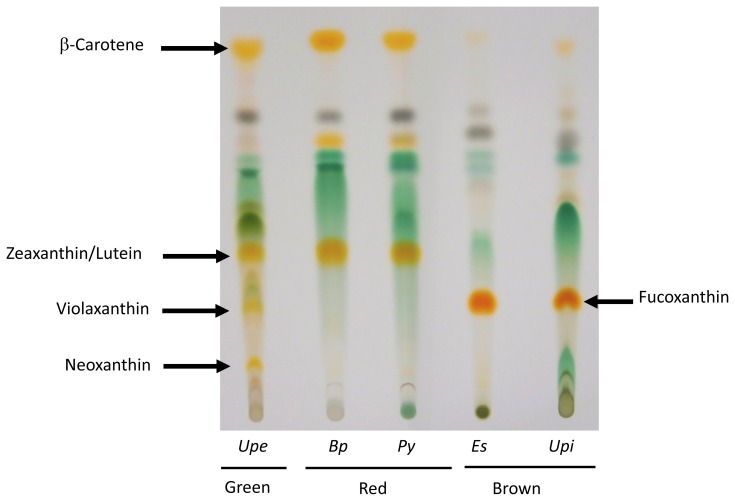
Thin-layer chromatography (TLC) analysis of carotenoids in seaweeds. Total lipids were extracted from the green seaweed, *Ulva pertusa* (Upe), red seaweeds, *Bangia fuscopurpurea* (Bf) and *Porphyra yezoensis* (Py), and brown seaweeds, *Ectocarpus siliculosus* (Es) and *Undaria pinnatifida* (Upi), with methanol. To detect each carotenoid contained in algae, total lipids were developed on a silica gel TLC plate with petroleum ether: acetone (7:3, *v*/*v*). We have confirmed that violaxanthin and fucoxanthin can be distinguished by UV-Vis spectrum.

Carotenoids are usually divided into two classes: carotenes and xanthophylls; the latter contains oxygen-containing functional group in its molecular structure. The distribution and color profiles of carotenes and xanthophylls are analyzed by thin-layer chromatography (TLC), although more precise or quantitative analysis requires high-performance liquid chromatography (HPLC). An example of a comparison of carotenoid content in seaweeds by TLC is shown in Figure 2. The chromatogram clearly demonstrates that seaweeds contain class-specific compositions of xanthophylls. For example, red seaweeds contain mainly zeaxanthin and lutein [17–19], whereas fucoxanthin is the major xanthophyll in brown seaweeds [14–16]. Green seaweed contains xanthophylls, such as zeaxanthin, violaxanthin and neoxanthin, as found in terrestrial green plants [20].

**Figure 3:**
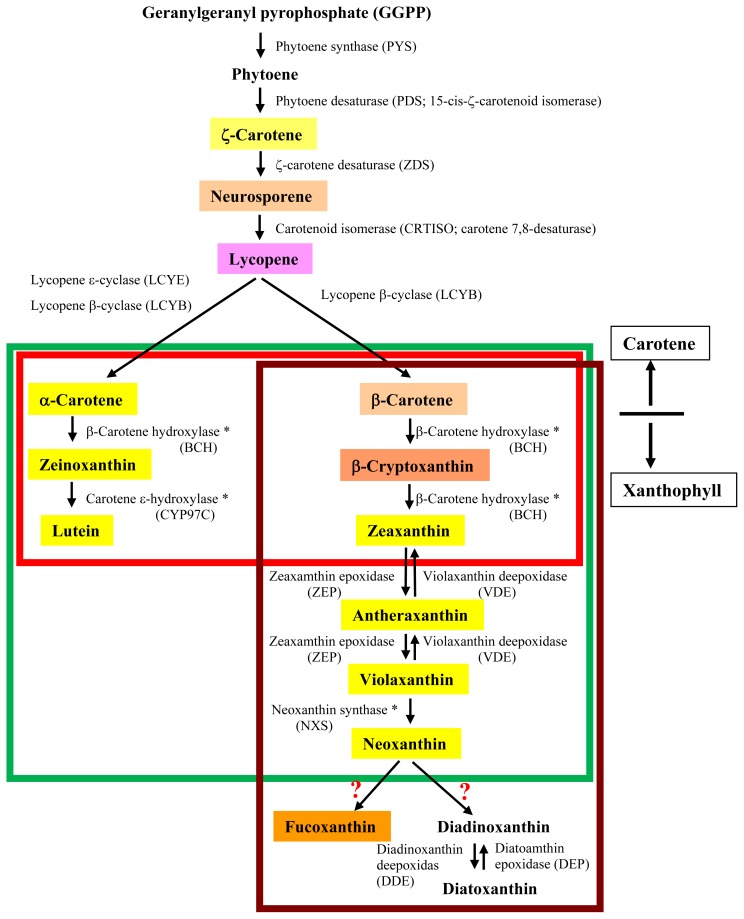
Predicted carotenoid biosynthetic pathway in seaweeds. Red, green and brown boxes reveal the pathway in red, green and brown seaweeds, respectively. The pathways for biosynthesis of zeaxanthin and lutein in red seaweeds and neoxanthin in green seaweeds are proposed based on genomic sequences from *Cyanidioschyzon merolae* and *Chlamydomonas reinhardtii*. The color overlapping the name of each carotenoid corresponds to its visible color. The symbol * indicates enzymes unidentified in seaweeds.

Figure 3 shows the biosynthetic pathway of carotenoids, which is based on the pathways known to operate in terrestrial green plants [1,3,4]. Carotenoid biosynthesis begins by head-to-tail condensation of two C_20_ geranylgeranyl pyrophosphate (GGPP) molecules to produce C_40_ phytoene by phytoene synthase (PYS). Then, phytoene is sequentially modified to ζ-carotene, neurosporene and lycopene by phytoene desaturase (PDS), ζ-carotene desaturase (ZDS) and carotenoid isomerase (CRTISO), respectively, increasing the number of conjugated carbon-carbon double bonds at each step. The terminal isoprene structures of the lycopene molecules are then cyclized by lycopene β-cyclase (LCYB) to produce β-carotene. As terrestrial green plants and all classes of seaweeds contain β-carotene, the biosynthetic pathway of β-carotenes seems to be conserved among these organisms. Indeed, genes encoding GGPP, PDS, ZDS, CRTISO and LCYB are found in algae and terrestrial plants.

The distribution of xanthophylls is, however, class-specific (Figure 2). Boxes colored green, red or brown in Figure 3 indicate the biosynthetic pathway for xanthophylls in green, red or brown seaweeds, respectively. The green box corresponds to both green algae and terrestrial plants containing xanthophylls derived from both α- and β-carotene [20]. Red seaweeds generally lack the xanthophyll biosynthesis pathway after zeaxanthin, which results in accumulation of zeaxanthin and lutein as major carotenoids, as seen in Figure 2[17–19]. This means that, like cyanobacteria, red seaweeds lack the xanthophyll cycle, known as the violaxanthin cycle [21], a reversible sequential conversion of zeaxanthin, antheraxanthin and violaxanthin by epoxidation or de-epoxidation (see Figure 3), to control light absorption in photosynthetic machinery under various environmental stress conditions.

In contrast, as shown in Figure 3, brown seaweeds and diatoms contain fucoxanthin, as well as diadinoxanthin and diatoxanthin, both of which are rarely detected using TLC, in addition to the xanthophylls found in green terrestrial plants, such as antheraxanthin, violaxanthin and neoxanthin, but do not contain α-carotene derivatives [14–16]. Therefore, brown seaweeds have a form of the xanthophyll cycle, designated the diadinoxanthin cycle, a reversible interconversion of diadinoxanthin and diatoxanthin [21,22], in addition to the violaxanthin cycle (Figure 2). Because the lycopene α-cyclase (LCYE) gene seems to have arisen by gene duplication in an algal ancestor and brown algae originated from the secondary endosymbiosis of a red alga, brown seaweeds are thought to have lost the LCYE gene and recruited new genes for the biosynthesis of novel xanthophylls, such as fucoxanthin, diadinoxanthin and diatoxanthin. However, the evolutionary origins of these genes have not been conclusively determined.

## 3. Putative Biosynthetic Pathway of Fucoxanthin in Brown Seaweeds

There is much confusion regarding the biosynthetic pathway of fucoxanthin by competing hypotheses, as mentioned below. However, now that whole genome sequence data has been published for fucoxanthin-producing algae, like the brown seaweed, *Ectocarpus siliculosus* [23], and the diatoms, *Thalassiosira pseudonana* and *Phaeodactylum tricornutum* [24,25], we can compare the carotenogenic genes in these algae with those in red and green algae and terrestrial plants. Based on the currently available genome information, we propose a hypothetical fucoxanthin-biosynthetic pathway in brown seaweeds.

### 3.1. Proposed Pathways for Fucoxanthin Biosynthesis Based on Knowledge in Diatoms

**Figure 4:**
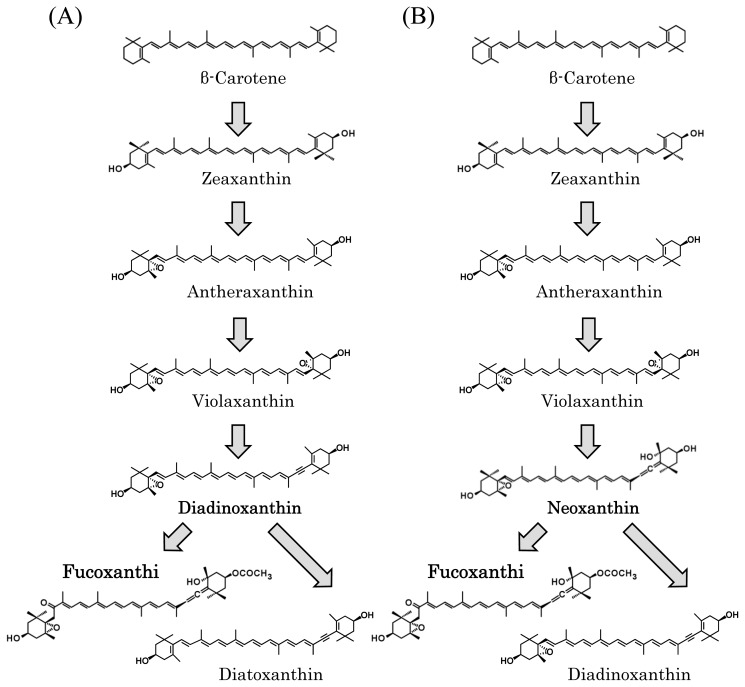
Two different hypotheses regarding the biosynthetic pathways for fucoxanthin. (**A**) Diadinoxanthin hypothesis: β-carotene is converted to fucoxanthin and diatoxanthin from diadinoxanthin; (**B**) Neoxanthin hypothesis: β-carotene is converted to fucoxanthin and diadinoxanthin from neoxanthin.

Diatoms, unicellular microalgae enclosed in a silicaceous frustule, produce fucoxanthin, although the biosynthetic pathway for this xanthophyll is unknown in these phytoplanktons. According to genome analysis of two species, *T. pseudonana* and *P. tricornutum* [24,25], two different pathways are proposed, which we have designated the diadinoxanthin hypothesis and the neoxanthin hypothesis (Figure 4A,B, respectively). The diadinoxanthin hypothesis involves a sequential conversion of violaxanthin to diadinoxanthin, which is a precursor of fucoxanthin [14,15,22]. The neoxanthin hypothesis, on the other hand, proposes a branching of the pathway from neoxanthin to both diadinoxanthin and fucoxanthin [2,16]. The latter hypothesis completely supports our proposed pathway for brown seaweeds; that is, we have also proposed two derivatives, diadinoxanthin and fucoxanthin, from neoxanthin, as shown in Figures 3 and 4B. Despite differences in these hypotheses, the biosynthetic pathway from β-carotenoid to violaxanthin is common to both diatoms and brown algae (Figure 3), because genes encoding zeaxanthin epoxidase (ZEP) and violaxanthin de-epoxidase (VDE) are conserved in these organisms (Table 1). Therefore, the reasons for these differing hypotheses regarding the fucoxanthin biosynthetic pathways are: (1) that no pathway intermediate has been detected by HPLC, and (2) that the genes encoding enzymes involved in the biosynthesis of fucoxanthin have not been cloned. For conversion of neoxanthin to fucoxanthin, two sequential reactions are necessary: ketolation of neoxanthin and acetylation of an intermediate [16]. Thus, biochemical detection of the intermediate, which is probably fucoxanthinol, and identification of genes encoding ketolase and acetylase are necessary to support the neoxanthin hypothesis for brown seaweeds.

### 3.2. Unique Features of Carotenogenic Genes

Genome analysis of diatoms revealed genes for enzymes similar to VDE, designated violaxanthin de-epoxidase-like (VDL), whose C-terminal domain is uncharged, in contrast to the Glu-rich *C*-terminus of VDE. In fact, genes for VDL are found in diatoms, dinoflagellates and a brown algae, *Ectocarpus siliculosus* (Table 1). Thus, Coesel *et al.* [15] hypothesized the involvement of VDL in de-epoxidation of the brown algae-specific xanthophyll, diadinoxanthin, to produce fucoxanthin.

It is worth noting that *Chlamydomonas reinhardtii* has a VDE-related (VDR) gene, but no genes for VDE or VDL, as shown in Table 1[15]. Although VDR lacks the Glu-rich domain, it is possible that VDR functions similarly to VDE in *C. reinhardtii*. However, green terrestrial plants and brown algae that contain genes for VDE also have genes for VDR (Table 1), which suggests functional similarity of VDE and VDR in general. The function of VDR is still unclear.

The *C*-terminal region of ZEP in diatoms and *E. siliculosus* contains no forkhead-associated (FHA) domain, which is generally conserved among ZEPs found in terrestrial plants [15], suggesting a specific role for this region in brown algae. Moreover, multiple ZEP genes are found in diatoms: two copies exist in *T. pseudonana* and three copies are found in *P. tricornutum* (Table 1). Thus, the involvement of a particular ZEP isoform in the diadinoxanthin cycle is proposed, as in the case for VDL. However, our database search indicated that *E. siliculosus* has only a single copy of the ZEP gene in its nuclear genome, as in terrestrial green plants, which raises the question of whether the diadinoxanthin cycle exists in brown seaweeds. This is the major issue for our proposal that fucoxanthin is biosynthesized from neoxanthin in brown seaweeds. Indeed, diadinoxanthin and diatoxanthin are difficult to detect by biochemical approaches in *E. siliculosus* (data not shown), although these xanthophylls have been detected in the diatom *P. tricornutum* by HPLC [16].

### 3.3. The Absence of Genes Encoding β-Carotenoid Hydroxylase and Neoxanthin Synthase

Although the neoxanthin hypothesis (Figure 4B) is simple, there are two problems that should be resolved. First, brown seaweeds lack a gene encoding β-carotenoid hydroxylase (BCH), as shown in Table 1. Genes for BCH are designated as *crtR* in cyanobacteria and *crtZ* in green algae and terrestrial plants. We performed nucleotide homology searches against the *E. siliculosus* genome, but no homologue for either *crtR* or *crtZ* was found in this brown seaweed. Recently, similar results were reported for red *Porphyra* species, for which large-scale EST databases have been established [17]. Moreover, although the chloroplast genome of the unicellular red alga, *Cyanidioschyzon merolae*, contains a gene encoding a CrtR-type BCH [18], there are no *crtR*- and *crtZ*-type genes in the *Porphyra* or *E. siliculosus* chloroplast genomes. Thus, it is possible that brown and red seaweeds produce zeaxanthin using an as yet unidentified BCH that may be structurally unrelated to the CrtR and CrtZ proteins. In this respect, the enzyme, carotene ɛ-hydroxylase-like (lutein deficient-like, LTL), has been hypothesized to function as a BCH in diatoms [14,15], as there are two copies of these genes (Table 1) and no α-carotene in diatoms. However, homology searches indicated that the *E. siliculosus* genome contains no homologue of LTL, suggesting that the novel protein with BCH-like activity is not related to LTL in brown seaweeds.

Second, brown seaweeds lack a gene encoding neoxanthin synthase (NXS) (Table 1), although it has been demonstrated that abscisic acid-deficient 4 (ABA4) is involved in the NXS activity in *Arabidopsis thaliana* [26]. The neoxanthin hypothesis is based on the presence of the NXS gene and neoxanthin (Figure 4B); however, neoxanthin has not been detected in brown seaweeds by biochemical analyses to date. As in the case for β-carotenoid hydroxylase, it is possible that brown seaweeds possess an unidentified NXS structurally unrelated to NXSs identified so far. Alternatively, because NXS and LCYB share 64% amino acid identity, an LCYB-like enzyme probably catalyzes the production of neoxanthin. However, the *E. siliculosus* genome contains only a single copy of the LCYB gene, suggesting the absence of an LCYB-like NXS in brown seaweeds. Identification of a new type of NXS gene would therefore be important to support the neoxanthin hypothesis.

### 3.4. Unknown Ketolase Involved in Fucoxanthin Biosynthesis

In contrast to the current understanding of the roles of VDE and ZEP, little is known about enzymes involved in fucoxanthin biosynthesis from neoxanthin or violaxanthin. As mentioned above, the synthesis of fucoxanthin requires ketolation of neoxanthin (Figure 3B), although the nature of the ketolase involved in this reaction is unclear. Thus, it is appropriate to consider the proposed biosynthetic pathway for the pink carotenoid, astaxanthin, which includes an oxygen-dependent introduction of keto-groups to β-carotene and zeaxanthin by CRTO/β-carotene ketolase (BKT) [27,28]. Functional expression of *C. reinhardtii* BKT increased the astaxanthin content of transgenic tobacco and *A. thaliana* plants [27,28]. Although the position of oxygenation differs between astaxanthin and fucoxanthin, it is possible that the amino acid sequences of ketolases targeting these two xanthophylls might be similar, particularly in their catalytic domains. Thus, we used green algal BTK sequences to perform homology searches against the *E. siliculosus* genome, but detected no homologues. Therefore, an unidentified ketolase specific to neoxanthin, in addition to an acetylase targeting fucoxanthinol, should be identified to understand the biosynthetic pathway of fucoxanthin in brown seaweeds.

### 3.5. Toward Resolution of the Fucoxanthin Biosynthetic Pathway in Brown Seaweeds

As mentioned above, homology searches using known carotenogenic genes have not been informative, which suggests that novel unknown genes are involved in fucoxanthin biosynthesis, for which experimental identification systems should be developed. The simplest approach is to screen for these genes in a heterologous genetic background, like *C. reinhardtii* or *Escherichia coli*. Because *C. reinhardtii* contains neoxanthin [29,30], changes in colony color could be useful for selection of *C. reinhardtii* transformed with a plasmid cDNA library derived from *E. siliculosus* mRNA or that of other brown seaweeds. Similarly, *E. coli* engineered to produce neoxanthin would be useful for screening cDNA libraries for fucoxanthin biosynthesis genes based on changes in colony color. However, in one study, the genes for ZEP from *P. tricornutum* did not produce active enzymes in *E. coli* [16], suggesting a difficulty in employing *E. coli* for our purpose. Alternatively, a collection of color mutants would be another way to identify genes for fucoxanthin biosynthesis, especially for *E. siliculosus*, for which there is now a complete genome sequence. However, mutations in steps of carotenoid biosynthesis that are already known would also result in color changes in seaweeds, which could be a difficulty for selection of knock-out mutants in the pathway downstream of neoxanthin or violaxanthin. Taken together, functional cloning using *C. reinhardtii* by color selection seems to be the best method for identifying the target carotenogenic genes.

## 4. Health Benefits of Fucoxanthin

### 4.1. Antioxidant Activity

Antioxidant activity is an important function in the body, as dysfunction of the antioxidant defense system leads to excessive oxidative stress. Recently, oxidative stress has been reported to be involved in the pathogenesis of several diseases, including cardiovascular disease, and natural antioxidants have received much attention in the prevention of disease [31]. Carotenoids have many physiological and biological functions, including their antioxidant properties, such as quenching of singlet oxygen and radical scavenging [32], which may help to maintain health and prevent disease.

Fucoxanthin has been reported to effectively scavenge chemically-generated free radicals, such as DPPH (1,1-diphenyl-2-picrylhydrazyl) [33]. Furthermore, fucoxanthin and its metabolite, fucoxanthinol, displayed antioxidant activities attributed to scavenging free radicals and quenching singlet oxygen *in vitro* [34]. The hydroxyl radical scavenging activities of fucoxanthin and fucoxanthinol were 13.5- and 1.7-times higher than that of α-tocopherol, but the singlet oxygen-quenching ability of fucoxanthin and fucoxanthinol was lower than that of β-carotene, with quenching rate constants (*k*_Q_) being 1.19, 1.81 and 12.78 × 10^10^ M^−1^ s^−1^ for fucoxanthin, fucoxanthinol and β-carotene, respectively. Interestingly, fucoxanthin acts as an antioxidant under anoxic conditions, whereas other carotenoids, such as β-carotene and lutein, show little or no quenching activities in such chemical assessment systems [35].

### 4.2. Anti-Obesity and Anti-Diabetic Effects in Animals

Obesity has increased drastically in recent years and is a major risk factor for type 2 diabetes, hyperlipidemia and hypertension [36]. The cluster of these diseases, known as metabolic syndrome, has become a worldwide problem. In obesity, dysregulation of adipocytokine production in white adipose tissue (WAT) is induced through excessive fat accumulation and induces insulin resistance, which leads to type 2 diabetes [37].

**Figure 5:**
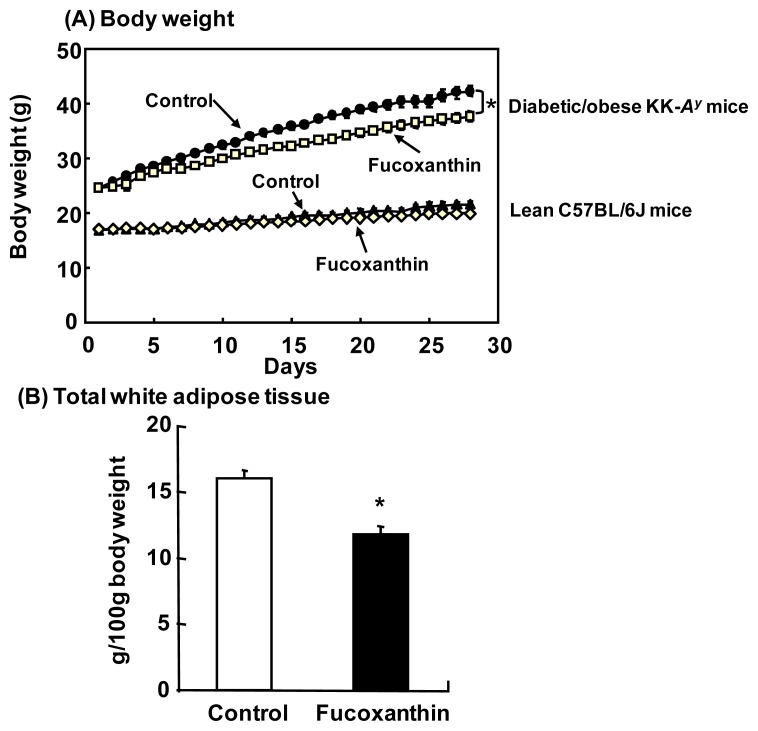
Anti-obesity effects of fucoxanthin on diabetic/obese mice. (**A**) Body weight of diabetic/obese KK-*A^y^* mice and lean C57BL/6J mice after four weeks of feeding of diets with/without 0.2% fucoxanthin; (**B**) White adipose tissue weight of KK-*A^y^* mice fed the diet with 0.2% fucoxanthin for two weeks. *p < 0.05 compared with controls.

We reported that dietary fucoxanthin attenuated both body weight and WAT weight gain in diabetic/obese KK-*A^y^* mice, but did not affect these parameters in lean C57BL/6J mice [38] (Figure 5). It is noteworthy that fucoxanthin induces mitochondrial uncoupling protein 1 (UCP1) in the WAT of obese mice [39]. UCP1 is typically expressed in brown adipose tissue (BAT) and promotes energy expenditure by thermogenesis [40], but is usually expressed only at low levels in WAT. Recently, apart from classic brown adipocytes present in BAT, brown-like adipocytes (termed “bright” or “beige” adipocytes) expressing UCP1 have been observed in WAT depots upon cold exposure or β-adrenergic stimulation [41,42]. These adipocytes can turn on a robust program of mitochondrial respiration and energy expenditure similar to that of brown adipocytes [43,44]. Thus, the anti-obesity effects of fucoxanthin may be related to the browning of white adipocytes through upregulation of UCP1, which results in increased energy expenditure in the body.

Moreover, fucoxanthin exhibited anti-diabetic activities in diabetic/obese KK-*A^y^* mice [45] and normal mice fed a high-fat diet [46]. Blood glucose levels were markedly reduced by the activation of glucose transporter 4 through improving insulin resistance in skeletal muscle of KK-*A^y^* mice [47]. In WAT of KK-*A^y^* mice fed fucoxanthin, mRNA expression levels of pro-inflammatory adipocytokines, such as interleukin-6 (IL-6) and tumor necrosis factor-α (TNF-α), which are thought to induce insulin resistance, were markedly suppressed [38]. Therefore, a potential mechanism for the anti-diabetic effect of fucoxanthin could be at least partly due to the improvement of insulin sensitivity through downregulation of pro-inflammatory adipocytokines.

In addition to the effects demonstrated in animal studies, Abidov *et al.* [48] recently showed that a mixture (Xanthigen) of fucoxanthin and pomegranate seed oil reduced body weight, body fat and liver fat content in either obese, non-diabetic premenopausal women diagnosed with non-alcoholic fatty liver disease or women with normal liver fat in a 16-week clinical trial. From these results, fucoxanthin is expected to be useful for the prevention of obesity, type-2 diabetes and metabolic syndrome.

### 4.3. Anti-Cancer Effects

Cancer is a major public health problem worldwide. The failure of conventional chemotherapy, in particular, to reduce mortality rates for carcinomas of the lung, colon, breast and prostate indicates a need for new approaches to prevent cancer and control its development [49]. One promising approach is chemoprevention, a nutraceutical and pharmacological approach to suppress or prevent the progression of carcinogenic processes to neoplastic disease. A number of naturally occurring compounds, particularly antioxidative compounds, including carotenoids, have shown chemopreventive activity [50]. In addition, dietary carotenoid intake has been correlated with reduced cancer [51], although several large-scale intervention trials using β-carotene failed to find chemopreventive effects [52–54]. Recently, however, several naturally occurring carotenoids other than β-carotene, including fucoxanthin, have exhibited chemopreventive or anticancer effects.

Anti-cancer effects of fucoxanthin are summarized in Table 2. Fucoxanthin inhibited proliferation of hepatoma HepG2 cells [55] and colon cancer Caco-2, HT-29 and DLD-1 cells *in vitro* [56]. The induction of apoptosis and suppression of cyclin D levels are proposed mechanisms for the observed anti-proliferative effect of fucoxanthin. These anticancer effects of fucoxanthin were stronger than those of β-carotene. Further, fucoxanthinol, which is a metabolite of fucoxanthin [57], also showed higher apoptosis-inducing activity on Caco-2 (colon) and MCF-7 (breast) cancer cells compared to fucoxanthin [58] (Table 2). These results indicate that dietary fucoxanthin is converted to a carotenoid, fucoxanthinol, with high potential as an anti-cancer agent in the body.

During *in vivo* studies, fucoxanthin was found to inhibit mouse colon carcinogenesis induced by 1,2-dimethylhydrazine [59] (Table 2). In addition, fucoxanthin has been reported to inhibit duodenal and skin carcinogenesis and liver tumorigenesis in mice. These anti-cancer effects of fucoxanthin are thought to operate by apoptosis induction [60], cell cycle arrest [61] and antioxidant activity [62]. However, the molecular mechanisms of the anti-cancer effects of fucoxanthin *in vivo* remain unknown. Further investigation using animal models is needed to clarify the mechanisms of the chemopreventive effects of fucoxanthin for different types of cancer.

### 4.4. Future Perspectives

The multifunctional nature of fucoxanthin encourages its development and use as a nutraceutical [12,63]. Fucoxanthin was also shown to be nontoxic in a mouse model [64], while it increased both serum HDL and non-HDL cholesterol levels [65]. However, the only study in humans [48] tested an algal extract product containing fucoxanthin, rather than purified fucoxanthin, together with pomegranate seed oil containing conjugated linolenic acids. For fucoxanthin to become useful in the medical and nutraceutical fields, more human studies, including clinical trials, will be needed to test for the effects of fucoxanthin on human health.

In experiments in mice testing for anti-obesity and anti-diabetic effects, an intake of more than 100 mg fucoxanthin/kg body weight (feeding 0.1% fucoxanthin-containing diet) for four weeks was not sufficient to exhibit any benefits [38,45]. On the other hand, Abidov *et al.* [48] found that dietary administration of 2.4 mg fucoxanthin per day (average body weight of volunteers was 100 kg) increased energy expenditure in the body and resulted in significant weight loss after 16 weeks. Thus, the amount of fucoxanthin necessary to exhibit an anti-obesity effect could be very different between mice and humans. Therefore, an effective dose and formulation of fucoxanthin for each aspect of health should be defined for human utilization of fucoxanthin as a nutraceutical. Of course, the mechanisms responsible for any differences in the effectiveness of fucoxanthin between rodents and humans should be investigated.

The molecular mechanisms of the anti-cancer effects of fucoxanthin could be partly due to the induction of apoptosis and cell cycle arrest in cancer cells. To fully investigate the molecular mechanisms of anticancer effects in cell culture experiments, fucoxanthin metabolites, such as fucoxanthinol and amarouciaxanthin A, should be included, because dietary fucoxanthin is converted to fucoxanthinol and amarouciaxanthin A in mice [57]. Fucoxanthinol has also been detected in the serum of humans after fucoxanthin administration [66]. Further investigation is required to assess the molecular mechanism of fucoxanthin against different types of cancer using animal models and human cell lines.

## 5. Conclusions

Growing evidence from animal studies shows that fucoxanthin has great potential in the prevention of diseases or management of human health. Despite such great progress in the characterization of its potential health-promoting activities, the biosynthetic pathway of fucoxanthin in brown seaweeds is not yet fully understood. To exploit our knowledge regarding this carotenoid in the medical and nutraceutical fields, resolution of this pathway at the molecular level is very important, because carotenogenic genes and carotenoids are highly useful for industrial applications. Thus, identification of genes involved in fucoxanthin production in a heterologous *C. reinhardtii* background will be fundamental for both basic biological and medical studies. Importantly, as this approach is also applicable to diatoms, fucoxanthin biosynthesis genes identified in a diatom system should be comparable with those in brown seaweeds, according to the hypotheses shown in Figure 4. Therefore, parallel progress in studies of novel genes in both brown seaweeds and diatoms would be ideal for understanding fucoxanthin biosynthesis and should, in turn, stimulate molecular biological and applied studies of the health benefits of fucoxanthin.

## Figures and Tables

**Table 1:** Comparison of carotenogenic genes involved in xanthophyll biosynthesis.

	BCH	LTL	ZEP	VDE	VDL	VDR	NXS
Brown algae							
*E. siliculosus*	−	−	+	+	+	+	−
*T. pseudonana*	−	++	++	+	+	+	−
*P. tricornutum*	−	++	+++	+	++	+	−
Red algae							
*C. merolae*	+ (Chl)	−	−	−	−	−	−
*P. umbilicalis*	−	−	+	−	−	−	−
*P. purpurea*	−	−	+	−	−	−	−
Green alga							
*C. reinhardtii*	+ (Partial)	−	+	−	−	+	−
Terrestrial plant							
*A. thaliana*	−	+++++	+	+	−	+	+

Symbols “+” and “−” represent presence or absence, respectively. The number of “+” indicates the copy number of genes present in the genome. The data for *P. umbilicalis* and *P. purpurea* were derived from NoriBLAST (http://dbdata.rutgers.edu/nori/) based on a large-scale EST analysis [17]. ZEP homologues were found in both *Porphyra* species, although their function is unknown. In *A. thaliana*, ABA4 is involved in the NXS activity [26]. LTL, lutein deficient-like; VDL, violaxanthin de-epoxidase-like; VDR, VDE-related; Chl, chloroplast genome.

**Table 2:** Anti-cancer effects of fucoxanthin and fucoxanthinol.

Carotenoid	Type of cancer	Mechanism	Target molecules	References
*In vitro*				
Fucoxanthin	GOTO (neuroblastoma)	G1 Cell cycle arrest	N-myc	[67]
	HL-60 (leukemia)	Apoptosis induction	Caspase-3, 7, 9	[68–70]
	Caco-2, HT29, DLD-1 (colon cancer)	Apoptosis induction	Bcl-2	[56]
	PC-3, DU-145, LNCap (prostate cancer)	Apoptosis induction	Bcl-2, Bax, Caspase-3	[71]
	DU-145, LNCap (prostate cancer)	G1 cell cycle arrest	GADD45A, SAPK/JNK	[72,73]
	HepG2 (hepato carcinoma)	G1 cell cycle arrest	Cyclin D	[55]
	SK-Hep-1 (hepato carcinoma)	G1 cell cycle arrest, apoptosis induction	Connexin 43, Connexin-32	[61]
	MGC-803 (gastric adenocarcinoma)	G2/M cell cycle arrest, apoptosis induction	Cyclin B1, Survivin	[74]
	EJ-1 (urinary bladder cancer)	Apoptosis induction	Caspase-3	[75]
	Caco-2 cell (colon cancer)	Enhancement on cytotoxicity of agents	MDR1	[76]
Fucoxanthinol	HL-60 (leukemia), MCF-7 (breast cancer), Caco-2 (colon cancer)	Apoptosis induction	Bcl-2	[58]
	PC-3 (prostate cancer)	Antiproliferative effect		[57]
	T cell leukemia	Antiproliferative effect		[77]
	BCBL-1, TY-1 (lymphoma)	G1 cell cycle arrest, apoptosis induction	NF-kB, AP-1, PI3kinase/Akt	[78]
*In vivo*				
Fucoxanthin	Colon cancer			[69]
	Liver tumorigenesis			[79]
	Duodenal carcinogenesis			[80]
	Sarcoma	Apoptosis induction	STAT3/EGFR	[60]
	Melanoma	Anti-melanogenesis	COX-2, p75NTR, EP1, MC1R	[81]
